# Is Obesity a Risk Factor for Vaccine Non-Responsiveness?

**DOI:** 10.1371/journal.pone.0082779

**Published:** 2013-12-11

**Authors:** Katherine M. Young, Clive M. Gray, Linda-Gail Bekker

**Affiliations:** 1 The Desmond Tutu HIV Centre, University of Cape Town, Cape Town, South Africa; 2 Division of Immunology, Institute of Infectious Disease and Molecular Medicine and Clinical Laboratory Sciences, Cape Town, South Africa; 3 National Health Laboratory Services, University of Cape Town, Cape Town, South Africa; South Texas Veterans Health Care System and University Health Science Center San Antonio, United States of America

## Abstract

Understanding the link between vaccine immunogenicity and efficacy is currently a major focus in HIV research. Consequently, recent developments in the HIV-1 vaccine field have led to a closer look at immune responses to known efficacious vaccines. We undertook a study to explore clinical predictors of vaccine efficacy following recombinant hepatitis B (rHBV) vaccination in a cohort of HIV-uninfected, hepatitis B virus naïve women living in a peri-urban setting in Cape Town. Our aim was to define host biological risk factors associated with lack of vaccine uptake. We found a significant association (p=0.009) between body mass index (BMI) and lack of vaccine-specific IgG titre (<10mIU/mL). Obese individuals (BMI ≥ 30kg/m^2^) were significantly more likely to be non-responders following 2 rHBV vaccine doses (Adjusted Odds Ratio of 8.75; p=0.043). There was no observed association between vaccine responses and age, method of contraception or time from vaccination to antibody measurement. These data suggest that obesity-associated factors interfere with vaccine immunogenicity and possible efficacy.

## Introduction

Two large HIV-1 vaccine trials have pointed to host factors being important in predicting vaccine efficacy. The Step Study, assessing the efficacy of the Merck Adenovirus type 5-vectored HIV-1 vaccine (MRKAd5), found Ad5 sero-positivity and lack of male circumcision to be associated with HIV-1 infection in men who have sex with men vaccine recipients [1], while the RV144 HIV vaccine trial conducted in Thailand demonstrated higher vaccine efficacy in individuals reporting lower risk sexual behaviour [2]. These associations suggest that pre-vaccination biological risk factors within the vaccine recipient might play a significant role in the generation of protective immune responses and that understanding these factors could be of importance in the development of an effective vaccine. By taking a “new” look at some “old” established efficacious vaccines, we may find clues as to how best to elicit a protective response to HIV-1 vaccines.

Hepatitis B Virus (HBV) in adults is predominantly sexually acquired and individuals at risk of infection are similarly at risk of acquiring HIV. The HBV vaccine is safe and effective with a known correlate of protection and has been widely used globally since its availability in 1982, with its inclusion in the South African Expanded Program of Immunization (EPI) schedule since 1995. The vaccine is a multi-dose, viral protein subunit vaccine containing HBV surface antigen (HBsAg) and an IgG response to this region is known to confer protection from infection. The outcomes of the RV144 HIV-1 vaccine trial in Thailand [2] and the subsequent immune-correlates analysis [3], suggest that an effective HIV vaccine will require a multi-dose, prime-boost regimen that elicits an IgG response to the V1V2 region of the *Env* protein. Our premise is that an rHBV vaccine could be used as a model to identify biological factors in vaccine recipients which might impact on efficacy and so provide insight into population variability which could be important for translating to the development of an HIV vaccine.

Several studies have been conducted in healthy populations in other parts of the world that have identified clinical predictors of a seroprotective response to rHBV. Estévez et al. showed that normal BMI (≤25kg/m^2^) and age <39 years were associated with higher seroprotection rates in healthy Cuban adults [4] and other studies have demonstrated that older age, male gender, obesity and smoking were associated with lower seroprotection rates [5,6] and lower HBsAb responses [7]. 

A robust and effective public health vaccine intervention programme to tackle the burden of infectious diseases in South Africa is crucial to maintain and expand particularly with regard to infections such as Tuberculosis (TB) and HIV. The parallel burden of non-communicable diseases, such as obesity [8,9], is also of concern, as this may be one biological factor which could impact on vaccine uptake and efficacy. As South Africa is poised to undertake large-scale HIV-1 vaccine efficacy trials, it is relevant to examine characteristics of vaccine volunteers that associate with vaccine non-responsiveness. Therefore, we asked whether obesity is associated with vaccine non-responsiveness using a known efficacious rHBV vaccine in a population likely to be involved in pending HIV vaccine trials.

## Methods

### Ethical Considerations

Study participants were identified from a database of potential participants for HIV prevention trials. All participants provided written informed consent prior to the collection of any participant data to be included in the database. Ethical approval for the study to establish the database was obtained from the Human Research Ethics Committee at the University of Cape Town. Data included in this analysis were extracted anonymously from the database. 

### Study Design and Participants

The study made use of existing data collected from HBV-naïve HIV uninfected women at high risk for HIV infection who had been vaccinated with the rHBV vaccine (Engerix-B^®^, GlaxoSmithKline) prior to being screened for an HIV pre-exposure prophylaxis study being conducted at the research centre. Women aged 18 years or older, living in a peri-urban setting outside Cape Town, who had received at least two of the three scheduled doses of Engerix-B^®^ at least 1 month apart and had anti-HBV surface antigen titres (HBsAb) measured at least 4 weeks after the second vaccination but prior to the third, were included in the study. All participants subsequently went on to receive the third dose of vaccine. HBsAb titres were measured using a standard validated serological assay and the reportable range for HBsAb titre was 10-1000mIU/mL. Participants with HBsAb titres below 10mIU/mL were reported as “<10mIU/mL” and those with titres above 1000mIU/mL were reported as 1000mIU/mL. HBsAb<10mIU/mL were assigned a value of 5mIU/mL for data analysis. A protective antibody titre (seroprotection) was defined as HBsAb≥10mIU/mL [10]. A detailed medical history was obtained for each participant prior to vaccination and a complete physical examination performed. No participants reported any acute illness at the time of vaccination.

### Statistical Considerations

The primary outcome variable was the presence or absence of a seroprotective vaccine response to the rHBV vaccine. We regarded this to be of greater clinical relevance than individual antibody titres to associate predictors of vaccine failure. Primary predictor variables included age, BMI, type of contraceptive used at baseline and time from administration of second vaccine dose to measurement of HBsAb titre. Reported co-morbid illnesses were considered for inclusion in the analysis. Descriptive statistics were computed for all variables as follows: for categorical variables, the number and percentage in each category; for continuous non-normally distributed variables, median, quartiles and range. None of the variables were normally distributed. Bivariate analyses were performed using Spearman’s correlation test statistic and the Mann-Whitney U-test for continuous and dichotomous variables respectively. Fisher’s exact test was used for variables with 3 or more categories. Multivariate logistic regression was used to adjust for multiple predictors of vaccine non-response. 

## Results

Of the 123 eligible participants, 25 (20%) had antibody titres <10mIU/mL and were deemed non-responders, while the remaining 80% were responders who demonstrated a seroprotective response [range: 10mIU/mL – upper limit of detection (1000mIU/mL)]. Participants were generally healthy with only 17 participants reporting any co-morbid chronic illnesses at baseline. Eight (8) participants had hypertension controlled with medication at baseline and 7 participants reported a history of non-severe asthma. Other reported conditions were allergic rhinitis (n=2), atopic eczema (n=2) and oral herpes simplex (n=1). Two (2) participants had both asthma and hypertension and 1 had asthma and atopic eczema. No participants had diabetes and none reported a history of previous hepatitis. No associations were observed between any reported chronic illness and vaccine response. 

**Figure 1:**
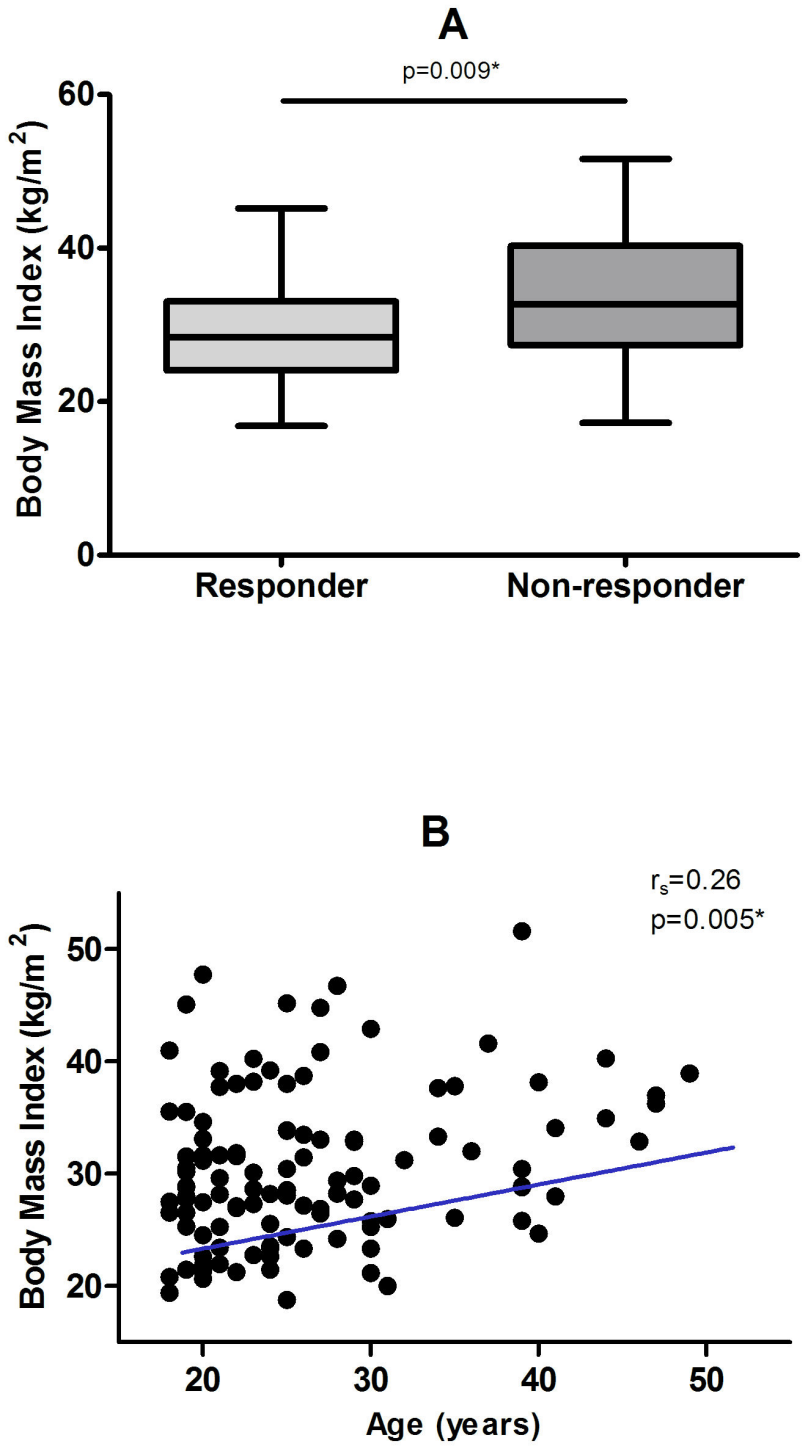
Associations of body mass index (BMI). (A) Box-and-whisker plot showing the relative distribution of BMI by vaccine response group (median, Interquartile range, minimum and maximum shown) (B) Spearman’s correlation of age at first vaccination and BMI.

Three quarters of the participants were under 30 years of age, with the oldest being 49 years old at the time of first vaccination. Time from second vaccination to measurement of HBsAb ranged from 29 to 314 days with the three quarters being tested within approximately 2 months (63 days) (Table 1). Neither age nor time to HBsAb measurement were significantly different between response groups (unadjusted p=0.15 and p=0.77 respectively, Table 1). No significant differences between contraceptive methods at baseline (classified as none/non-hormonal, oral or long-acting injectable contraceptives) were observed between response groups (Fisher’s exact test p=0.24, Table 1). A significant association was however identified between body mass index (BMI) and a seroprotective antibody response. Non-responders were seen to have significantly higher BMI than those who generated a protective response (p=0.009, table 1, Figure 1A). BMI was also seen to correlate weakly with age (Figure 1B) with older women being relatively heavier than younger women (r_s_=0.26; p=0.005). No significant relationships were observed between other predictor variables.

**Table 1:** Summary statistics by response group.

Variable	Responder		Non-responder		Mann-Whitney U-test
	N (%)	Median (IQR)	N (%)	Median (IQR)	
Age (years)	98 (80)	24 (20-29)	25 (20)	26 (22-34)	p=0.15
BMI (kg/m^2^)	98 (80)	28.40 (24.22-33.06)	24 (20)	32.66 (27.39-40.26)	p=0.009*
BMI Categories					P=0.035*^$^
• Underweight (<18.5 )	3 (75)	18.29 (16.81-18,37)	1 (25)	17.22 (N/A)	P=0.65
• Normal (18.5-24.9)	25 (96)	21.83 (21.26-23.37)	1 (4)	22.66 (N/A)	P=0.79
• Overweight (25-29.9)	30 (81)	27.73 (26.49-28.83)	7 (19)	27.30 (26.89-28.25)	P=0.76
• Obese (>30)	40 (73)	33.98 (31.64-38.19)	15 (27)	38.01 (33.31-45.09)	P=0.059
Days post vaccination	98 (80)	41 (33-62)	25 (20)	37 (34-66)	p=0.77
HBsAb titre (GMT / 95%CI)	98 (80)	72.61 (57.71-91.37)			
Contraception	98 (80)		25 (20)		
• None/non-hormonal	31 (89)		4 (11)		
• Oral combined pill	5 (83)		1 (17)		P=0.24^$^
• Long-acting injectable	61 (75)		20 (25)		

Data are presented as N (number of participants with each predictor variable in the respective response groups) and percentage of total with that predictor. BMI (Body Mass Index); HBsAb (Hepatitis B surface antibody); GMT (Geometric mean titre). IQR (Interquartile Range). ^*^ Illustrates p-value≤ 0.05. ^$^ Fisher’s exact test p-value. [Underweight category not included in calculation of Fisher’s exact test p-value due to the small sample size (n=4)].

When we divided participants into standard WHO defined BMI categories: Underweight (<18.5kg/m^2^), normal weight (18-5 – 24.9kg/m^2^), overweight (25 – 29.9kg/m^2^) and obese (≥30kg/m^2^), no significant differences in BMI were observed between response group within each category, however, obese non-responders had a tendency to higher BMI than responders (Table 1). The underweight category was excluded from this and all subsequent analyses given the small sample size (n=4). 

**Figure 2:**
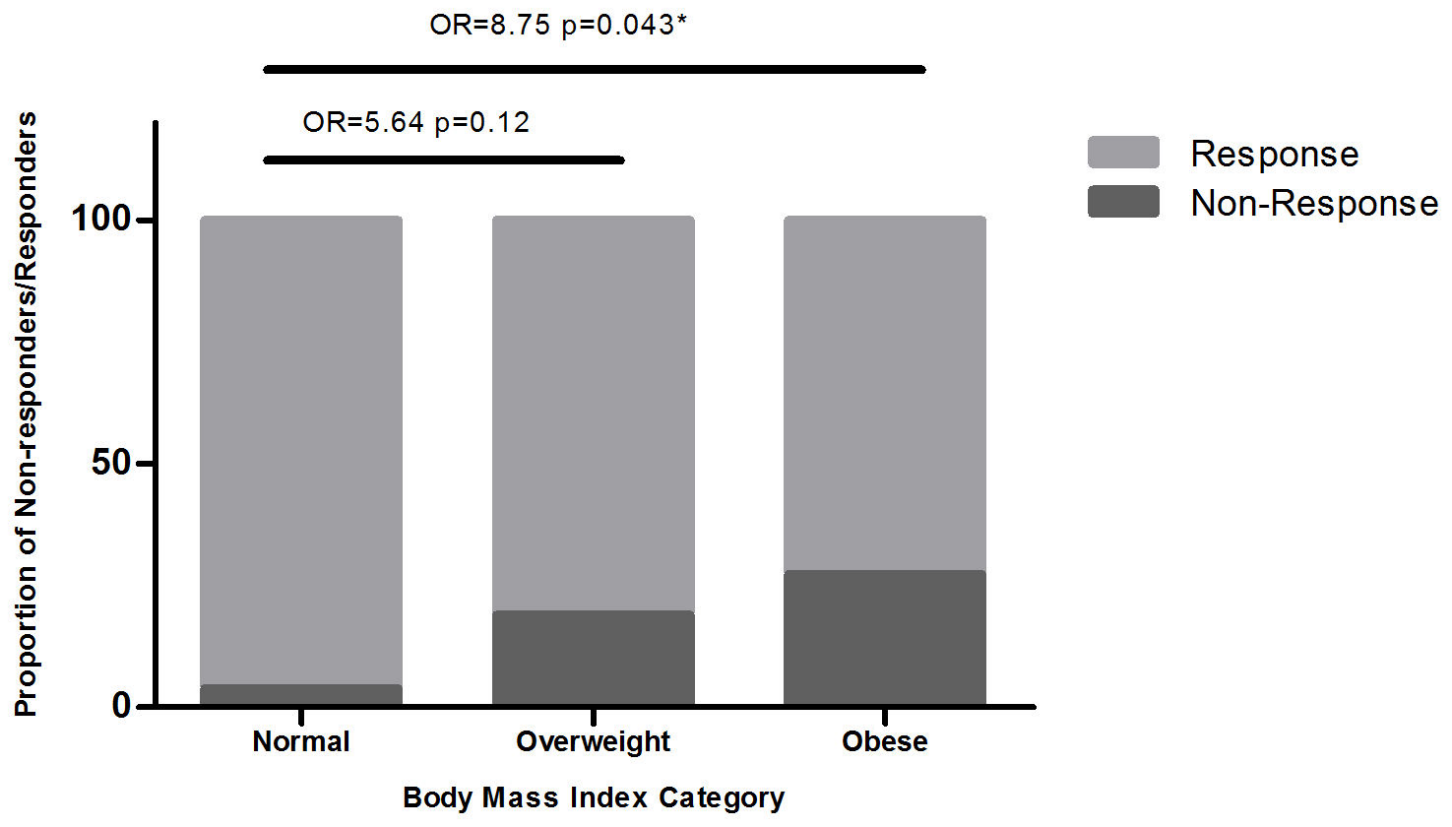
Relative distribution of vaccine non-response by body mass index (BMI) category. Illustrates the proportion of non-responders within each standard BMI category. The underweight category has been excluded as only 4 participants fell into this group.

Examination of the outcomes in terms of proportions of response revealed a significant difference in vaccine response between BMI categories (Fisher’s exact test; p=0.035). Individuals with normal BMI had a 96% response rate versus 81% and 73% in the overweight and obese groups respectively (Figure 2). 

Based on these data, we built two multivariate logistic regression models using forward selection, one included BMI as a continuous variable, the second evaluated relative differences between BMI categories. Age was included as a potential confounder although it did not significantly improve the model. No other possible predictors significantly improved either model. The final model therefore included age and BMI in either form (Table 2). From model 1 we see an 11% increase in odds of a non-response with each 1kg/m^2^ increase in BMI within our study population. Model 2 similarly demonstrated that obese individuals were significantly more likely to be non-responders following 2 rHBV vaccine doses (OR 8.75; p=0.043). Being in the overweight category was not associated with any difference in rate of seroprotection when compared with normal weight individuals (OR=5.64; p=0.12) (Figure 2). 

**Table 2:** Multivariate Analysis of predictors of non-response to recombinant hepatitis B vaccine.

Predictor	Odds Ratio (OR)	95% Confidence Interval	p-value
Model 1			
Age (years)	1.01	0.95 - 1.07	0.76
BMI**^*#*^** (kg.m^2^)	1.11	1.04 - 1.20	0.003^*^
Model 2			
Age (years)	1.02	0.96 - 1.08	0.60
Overweight relative to normal weight	5.64	0.65 - 49.20	0.12
Obese relative to normal weight	8.75	1.07 - 71.60	0.043^*^

Model 1 shows the logistic regression of vaccine non-response using BMI as a continuous variable^#^. Model 2 shows the same logistic regression if the BMI is categorised. BMI (Body Mass Index). ^*^Illustrates p-value≤0.05

## Discussion

This was a retrospective study to ask the question whether obesity was associated with vaccine efficacy, using a known efficacious rHBV, in a vaccine-volunteer population most likely to also participate in future HIV vaccine trials. It is known that four factors most commonly associate with vaccine non-responsiveness; age, BMI or obesity, male gender and smoking [4–7,10]. Although we only investigated for the effect of age and BMI of these previously described risk factors, we did assess the impact of hormonal contraceptives. Our data revealed no association with age, although this might be due to the relatively narrow age range of participants and the predominance of women under the age of 30 years. We also showed no link between vaccine response and contraceptive type at baseline. Our study was limited by the retrospective nature of the study design and the post hoc analysis of the data. 

Our finding that BMI, particularly over 30kg/m^2^ is associated with an increased risk of non-responsiveness to the rHBV vaccine is in agreement with findings from other populations [4–7]. A possible explanation for this phenomenon could be a distribution effect, in which case a different mode of delivery or different needle length may need to be used. A study in obese adolescents [11] showed that increasing the needle length in delivering the vaccine “rescued” a proportion of non-responders but did not account entirely for the lower response rates in obese individuals. An alternative possibility is that there could be more complex biological mechanisms at play and we speculate that there is a relationship between obesity, inflammation and vaccine immunogenicity. 

Obesity is increasingly being recognised as a low-grade chronic inflammatory condition [12–14] and it is possible that obese individuals exist in a pro-inflammatory state, which interferes with immunogenicity to vaccine candidates. The negative impact of obesity on vaccine immune outcomes have been shown for seasonal trivalent influenza vaccines (TIV) [15] and the tetanus toxoid (TT) vaccine [16]. It is also possible that differences in dietary intake may have an effect on the gastro-intestinal microbiota, and this in turn can affect the “leakiness” of the gut leading to bacterial translocation and increased levels of immune activation [17–20]. Whatever the mechanistic link between obesity, inflammation and vaccine non-responsiveness might be, the fact is that obesity is on the rise globally, and the 2003 South African Demographic and Health survey defined 55% of women and 30% of men as either overweight or obese based on BMI [9]. A later 2009/2010 survey commissioned by glaxosmithkline also associated a higher likelihood of being overweight or obese with being less affluent [21].

Understanding the mechanism underlying the relationship between obesity and immune response to vaccines is of importance in the development of all future vaccines including an effective HIV-1 vaccine. Additionally, efficacy of existing vaccines may be eroded as obesity increasingly becomes a problem. Further studies are obviously required to unravel this relationship. 
